# Examining the Post-operative Well-Being of Women Who Underwent Mammoplasty: A Cross-Sectional Study

**DOI:** 10.3389/fpsyt.2021.645102

**Published:** 2021-03-29

**Authors:** Jessica Ranieri, Fabiana Fiasca, Federica Guerra, Enrico Perilli, Antonella Mattei, Dina Di Giacomo

**Affiliations:** Department of Life, Health and Environmental Sciences, University of L'Aquila, L'Aquila, Italy

**Keywords:** aesthetic surgery, disease-related surgery, psychological adaptation, BREAST-Q, mental health

## Abstract

**Background:** Mammoplasty is the most common surgery used for breast augmentation (aesthetic plastic) and breast reconstruction (disease-related plastic) in women who have been diagnosed with and surgically treated for regional breast cancer with modified radical mastectomy. This study aims to examine the long-term effects of mammoplasty on the psychological well-being of women.

**Methods:** Participants were 44 women aged 30–50 years (mean = 40.4 ± 5.9). They were divided into two groups based on the purpose of the breast surgery they underwent [augmentation surgery (AS) vs. reconstruction surgery (RS)] and the time that had elapsed since their surgery (≤3 vs. >3 years).

**Results:** Our findings suggest that women who underwent AS reported a decline in their psychological well-being over time. The women who had undergone AS ≤3 and >3 years did not show any differences in emotional functioning, with the exception of the BREAST-Q scores on the satisfaction with breasts subscale. We examined the impact of mammoplasty on the satisfaction levels and well-being of women who had undergone RS (after MRM). They were less satisfied with their breasts than those who belonged to the AS group, confirming our hypothesis. However, this was true only among those who had undergone surgery ≤3 years earlier.

**Conclusions:** In conclusion, our findings underscore the need to provide psychological support to those who have undergone breast AS and RS. Additionally, this study implies the need for personalized psychological interventions to improve the emotional adaptation process and enhance women's mental well-being.

## Introduction

Mammoplasty is the most common surgery for breast augmentation, with about 280,692 healthy women having undergone aesthetic plastic surgery procedures in 2019 alone (1). Recently though, reports from The Aesthetic Society reveal a 14.9% decrease in breast augmentation surgery, which had been the number 1 aesthetic surgical procedure since 2017. Additionally, breast implant removal (explantation) increased by 34.4% (1). Several studies have already focused on aesthetic surgery, but they primarily paid attention to its technical aspects (2–7). Nava et al. (3) proposed that the measures adopted in augmentation surgery (AS) should be chosen based on an evaluation of the post-operative complications, reintervention rates, the reasons for reoperation, and patient-reported outcomes. Furthermore, they emphasized the need for a national register and mandatory reporting policies to identify and promote decisional pathways that yield better patient outcomes, in order to harmonize, implement, and maximize patient experience and usage of medical resources.

Several studies have examined the surgical outcomes of mammoplasty, while focusing on its technicalities. Over 40% of the women who underwent a mastectomy were for breast reconstruction or disease-related plastic surgery; these women had been diagnosed with and surgically treated for regional breast cancer using modified radical mastectomy (MRM) (2). In their review article, Coombs et al. (4) explored the literature on surgical procedures of breast augmentation and discussed the evolution of implants, patient assessments, and post-operative complications. Currently, aesthetic surgery offers innovative solutions that ensure the safety and well-being of women and are cognizant of their physical characteristics (5–7). Some studies in this domain have focused on the psychological and emotional effects of mammoplasty such as the psychological adaptation, sexual well-being, anxiety, quality of life, and body satisfaction/dissatisfaction (8–12). The effects of AS on the psychological and sexual well-being of women initially appear after 2–6 weeks of the surgery. Zaborsk et al. (8) found that breast AS does not affect neuroticism levels. This suggests that the constitutional personality traits of women who undergo such operations remain relatively unaffected after the surgical intervention. However, neuroticism may modulate the psychological changes that women experience after breast augmentation (e.g., higher post-operative life satisfaction). Coriddi et al. (9) reported a significant improvement in self-reported quality of life after breast AS. Changes in the levels of satisfaction with one's breasts, as well as their psychosocial and sexual well-being, were particularly significant. Swanson (10) adopted a psychological perspective and found that 91 and 64% of breast augmentations are successful in improving the individual's self-esteem and quality of life, respectively. Despite these positive outcomes, the researchers did not report the preoperative satisfaction levels; therefore, it is difficult to compare and comment on these results with reference to the baseline value.

Breast surgeries are also performed for disease-related purposes [i.e., as a reconstruction surgery (RS) after MRM for regional breast cancer]. A large population of women who choose to undergo breast augmentation surgeries for reconstructive surgery also have the desire to enhance their native breast volume. Patients who undergo breast RS after a mastectomy report greater satisfaction with surgical outcomes and a better body image than women who do not opt for reconstructive measures (10, 11). Most of the studies examining the outcomes of breast cancer treatment have focused on disease-free or overall survival and the effectiveness of therapeutic oncology regimens (12–14). The literature review implies that very few studies to date have examined the behavioral and psychological aspects associated with breast surgery (disease-related plastic surgery). Wehrens et al. (15) found that the psychological profiles of women who underwent RS are significantly better; they tend to be more extroverted, socially and sexually active, talkative, animated, and are more likely to take initiatives, revealing a positive emotional pattern in the early phases after a plastic surgery. In a study by Shekhawat et al. (16), such women reported higher levels of satisfaction (89%) with their post-reconstruction body image and overall satisfaction. Additionally, parameters such as anxiety and femininity showed a positive trend after a year of the RS. AS has been observed to result in more favorable aesthetic outcomes than breast RS. In previous studies, the outcomes of breast AS and RS have predominantly been examined within a short duration after the procedure. Some studies have examined temporal changes in the post-operative psychological variables (16). However, it is important to investigate the long-term positive and negative psychological consequences to better understand their emotional adaptation process.

This study aims to examine the long-term effects of mammoplasty on the psychological adaptation of women. Post-operative surveys were conducted to examine the changes in patient satisfaction and quality of life after breast surgery over a long period of time.

## Methods

### Ethical Statement

Ethical approval to conduct this study was granted by the Institutional Review Board of the University of L'Aquila, Italy (Prot. No. 36032/2017) and S. Salvatore Hospital of L'Aquila, Italy (the hospital from which the participants were recruited).

Informed consent was obtained from each participant, and the study adhered to the guidelines outlined in the Declaration of Helsinki (17).

### Participants

Forty-four women aged 30–55 years (mean = 40.4 ± 5.9 years) participated in this study. Those living in Italy were included in this study; additionally, their education levels were as follows: high school graduate (45.5%), graduate (34.5%), and not graduated (20.5%). Further, 70.5% of them were employed.

We contacted 56 eligible patients, and 44 of them provided informed consent. Twelve patients declined the invitation to participate in the study. The reasons for refusal were no need for support, lack of time, and waiting for caregiver. The participants were classified into two groups: (1) the RS group, consisting of 22 women who had undergone mammoplasty (post-mastectomy) after being diagnosed with breast cancer and (2) the AS group, consisting of 22 women who had undergone mammoplasty for aesthetic purposes. The women in the RS group were enrolled in the Oncological Division of S. Salvatore Hospital during their scheduled follow-up; women in the AS group of women had been enrolled in the hospital during their screening sessions for cancer prevention. The medical staff identified the eligible women; however, their participation was voluntary. Eligible participants met the following inclusion criteria: (a) age >18 years, (b) outpatients who have undergone mammoplasty, and (c) willingness to participate in the study and provide written informed consent. The exclusion criteria were (a) contraindications for mammoplasty (e.g., the presence of severe psychopathology, dysmorphic disorder, and severe systemic diseases), (b) cancer recurrence, (c) a prior diagnosis of cancer or concurrent diagnosis of another cancer, (d) mastectomy after breast cancer recurrence, and (e) the presence of severe chronic diseases or significant physical or psychological disabilities that could invalidate informed consent or their responses.

Table 1 presents the demographic characteristics of the participants.

**Table 1:** Demographic characteristics of the participants compared between the AS and RS groups.

	**Total** ***n* = 44**	**Surgical Groups**		***p***
		**AS*n*22 (50.00%)**	**RS*n*22 (50.00%)**	
**Age (years):** median (IQR)	40.5 (36.5–44.5)	39 (34–45)	42 (39–44)	0.158^*^
**Age groups**: *n* (%)				0.070^**^
≤40 years	22 (50.00)	14 (63.64)	8 (36.36)	
>40 years	22 (50.00)	8 (36.36)	14 (63.64)	
**Educational level**: *n* (%)				0.828^**^
Middle school	9 (20.45)	4 (18.18)	5 (22.73)	
High school	20 (45.45)	11 (50.00)	9 (40.91)	
University	15 (34.10)	7 (31.82)	8 (36.36)	
**Occupational status**: *n* (%)				0.195^**^
Unemployed	14 (31.82)	5 (22.73)	9 (40.91)	
Employed	30 (68.18)	17 (77.27)	13 (59.09)	
**Time since surgery**: *n* (%)				1.000^**^
≤3 years	28 (63.64)	14 (63.64)	14 (63.64)	
>3 years	16 (36.36)	8 (36.36)	8 (36.36)	
**BREAST-Q scales**				
**Satisfaction with breasts**: median (IQR)	52.5 (42–69.5)	59.5 (48–80)	47 (42–55)	0.019^*^
**Satisfaction with outcomes**: median (IQR)	75 (61–100)	88.5 (61–100)	71 (61–75)	0.216
**Psychological well-being**: median (IQR)	62.5 (48.5–89)	79 (62–93)	53 (43–63)	0.010^*^
**Sexual well-being**: median (IQR)	55 (31–65)	65 (53–65)	41 (22–60)	0.005^*^

* *Wilcoxon–Mann–Whitney test*.

** *χ^2^ test IQR, interquartile range; AS, augmentation surgery; RS, reconstructive surgery*.

### Sociodemographic Variables

Two types of data were collected. First, demographic information was collected using self-report measures. Of these, we included selected variables (e.g., educational level, occupation, and marital status) in the analysis, depending on whether they indicated the age or life stage. Second, clinical data were collected using psychological assessments.

### Assessments

The demographic characteristics of the participants were measured using a self-report questionnaire, and the BREAST-Q was used to evaluate the emotional impact of the mammoplasty.

#### Demographic Questionnaire (*ad hoc* Questionnaire)

This self-report measure was used to assess the demographic characteristics of the participants, such as age, educational level, occupational status, time elapsed since the mammoplasty, and the type of surgical intervention.

#### Italian Version of the BREAST-Q (Post-operative Version) (18)

The BREAST-Q assesses the patient-reported outcomes of aesthetic and reconstructive breast surgery (18). We implemented the relevant subscale for the participants of the AS and RS group; the scale is composed of four indices measuring the well-being and satisfaction: satisfaction with breasts, satisfaction with outcome, psychosocial well-being, and sexual well-being. All subscale scores range from 0 to 100 as per the Rasch model. Higher scores indicate greater satisfaction or better functioning.

### Procedure

This study was conducted in collaboration with general practitioners and breast cancer specialists. They identified eligible participants who were subsequently enrolled by appointments. Informed consent was obtained at the time of enrolment. Trained clinical psychologists, who were unaware of the study objectives, administered the psychological assessments in a quiet room within the hospital. The total duration of the evaluation procedure was 40 min. The data were collected anonymously.

### Study Design

The participants were divided into two groups depending on the type of breast surgery that they underwent (AS vs. RS) and the time that had elapsed since their surgery (≤3 vs. >3 years). Descriptive statistics were computed to examine the participant characteristics. For analyzing continuous variables (e.g., age, scores on the BREAST-Q scales), medians and their interquartile ranges (IQRs) were ascertained. The Wilcoxon–Mann–Whitney test was used to examine the differences between AS and RS groups. A chi-square test (χ^2^) was performed to compare the categorical variables (age ranges, geographical location, education levels, occupation, time after surgery), and the results were reported as counts and column percentages.

All statistical analyses were conducted using Stata. All the tests were two-tailed, and the statistical significance was set at *p* < 0.05.

## Results

**Figure 1:**
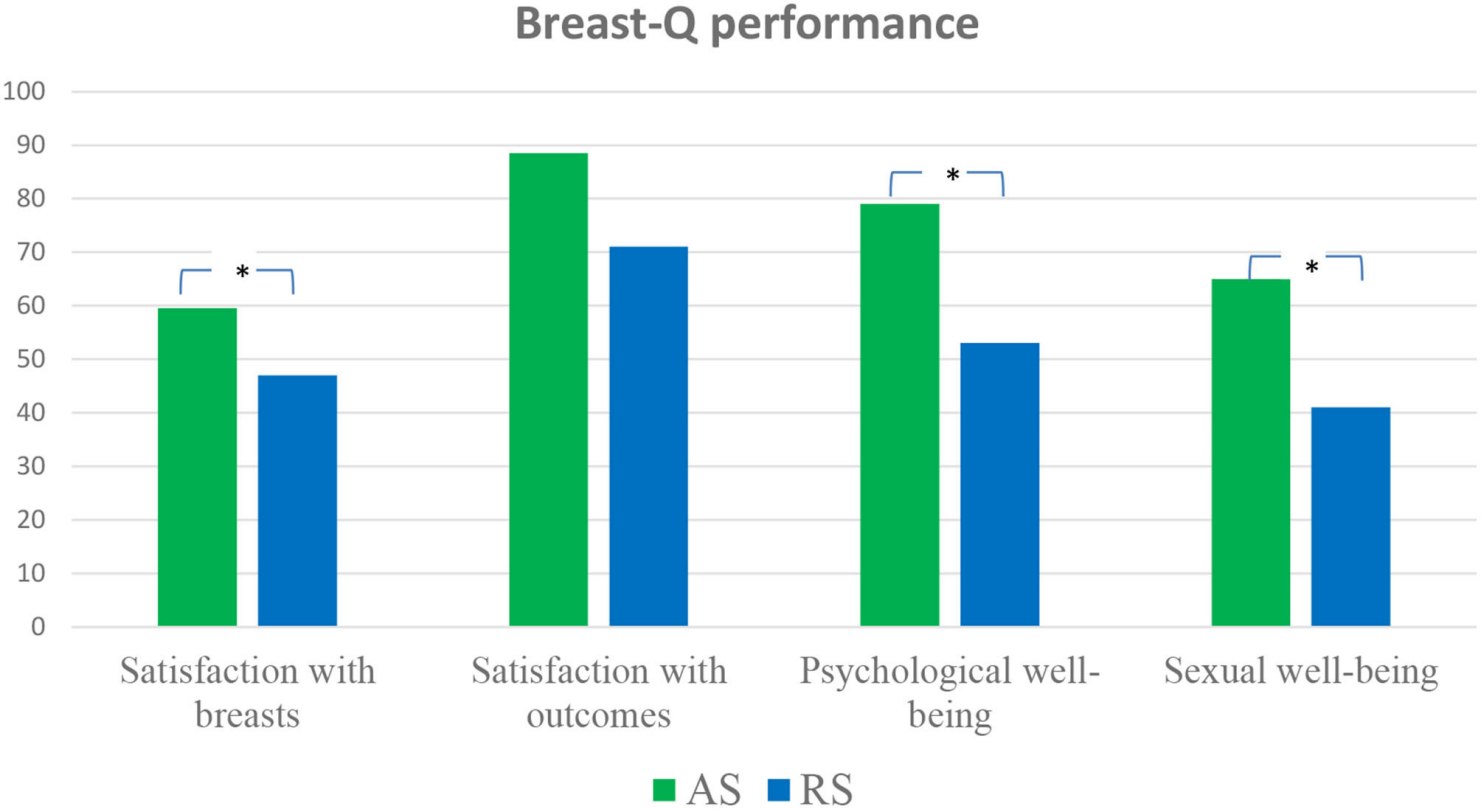
BREAST-Q indices of the AS and RS groups.

A total of 44 patients participated in this study. As shown in Table 1, their median age was 40.5 years [interquartile range (IQR): 36.5–44.5). The two age classes identified (≤40 and >40 years) were equally represented. More than half of the participants (*n* = 24, 54.55%) were from Central Italy, and almost half of them (*n* = 20, 45.45%) were high school graduates. Majority of them were employed (*n* = 30, 68.18%), and half of them worked as clerks (*n* = 15, 34.09%). Furthermore, 28 (63.64%) participants underwent surgery within the past 3 years. Median scores for BREAST-Q subscales were calculated: satisfaction with breasts (Med = 52.5, IQR = 42–69.5), satisfaction with outcome (Med = 75, IQR = 61–100), psychological well-being (Med = 62.5, IQR = 48.5–89), and sexual well-being (Med = 55, IQR = 31–65). Statistically significant differences were observed between the BREAST-Q scores of the AS and RS group participants (Table 1 and Figure 1).

The AS group participants obtained significantly higher scores (*x*) on the satisfaction with breasts (*x* = 59.5, *p* < 0.05), psychological well-being (*x* = 79, *p* < 0.05), and sexual well-being (*x* = 65, *p* < 0.01) subscales than the RS group participants (*x* = 47, 53, and 41, respectively).

BREAST-Q subscale scores were further compared on the basis of the time elapsed since the surgery (≤3 or >3 years) between the AS and RS groups (see Table 2). Scores on the satisfaction with breasts subscale were highest in the first 3 years after the surgery [*x* (≤3) = 59.5, *x* (>3) = 45.5, *p* < 0.05]. This difference was significant for the AS group [*x* (≤3) = 78.5, *x* (>3) = 52.5, *p* < 0.05]. Furthermore, participants from the AS group, who underwent surgery before ≤3 years, showed significantly higher scores on satisfaction with breasts (*x* = 78.5, *p* < 0.05) and sexual well-being (*x* = 65, *p* < 0.05) subscales compared to those in the RS group (*x* = 50, 41, respectively). However, those from the AS group who underwent breast surgery >3 years earlier (*x* = 73.5, *p* < 0.05) obtained significantly higher scores on psychological well-being subscale compared to those who belonged to the RS group (*x* = 56.5).

**Table 2:** BREAST-Q scores stratified by the time elapsed since surgery (≤3 years, >3 years) compared between the AS and RS groups.

	**Time since surgery**		p^*^
	**≤3 years*n*28 (63.64%)**	**>3 years*n*16 (36.36%)**	
**Satisfaction with breasts**: median (IQR)	59.5 (45.5–78.5)	45.5 (39.5–55.5)	**0.017**
AS	78.5 (58–85)	52.5 (44–57.5)	**0.029**
RS	50 (45–61)	42 (33–46.5)	0.055
p^*^	**0.023**	0.141	
**Satisfaction with outcomes**: median (IQR)	75 (63–100)	65 (57–75)	0.081
AS	100 (65–100)	65 (57–100)	0.360
RS	75 (61–100)	64 (52–71)	0.109
p^*^	0.270	0.597	
**Psychological well-being**: median (IQR)	60 (48.5–92)	68.5 (47.5–79)	0.769
AS	83.5 (58–100)	73.5 (70–85.5)	0.515
RS	52.5 (48–60)	56.5 (38–65)	0.973
p^*^	0.088	**0.036**	
**Sexual well-being**: median (IQR)	55 (29–65)	55 (35.5–65)	0.819
AS	65 (53–65)	61.5 (51–65)	0.730
RS	41 (22–60)	42 (25–58.5)	0.810
p^*^	**0.016**	0.138	

* *Wilcoxon–Mann–Whitney test*.

*IQR, interquartile range; AS, augmentation surgery; RS, reconstructive surgery*.

**Figure 2:**
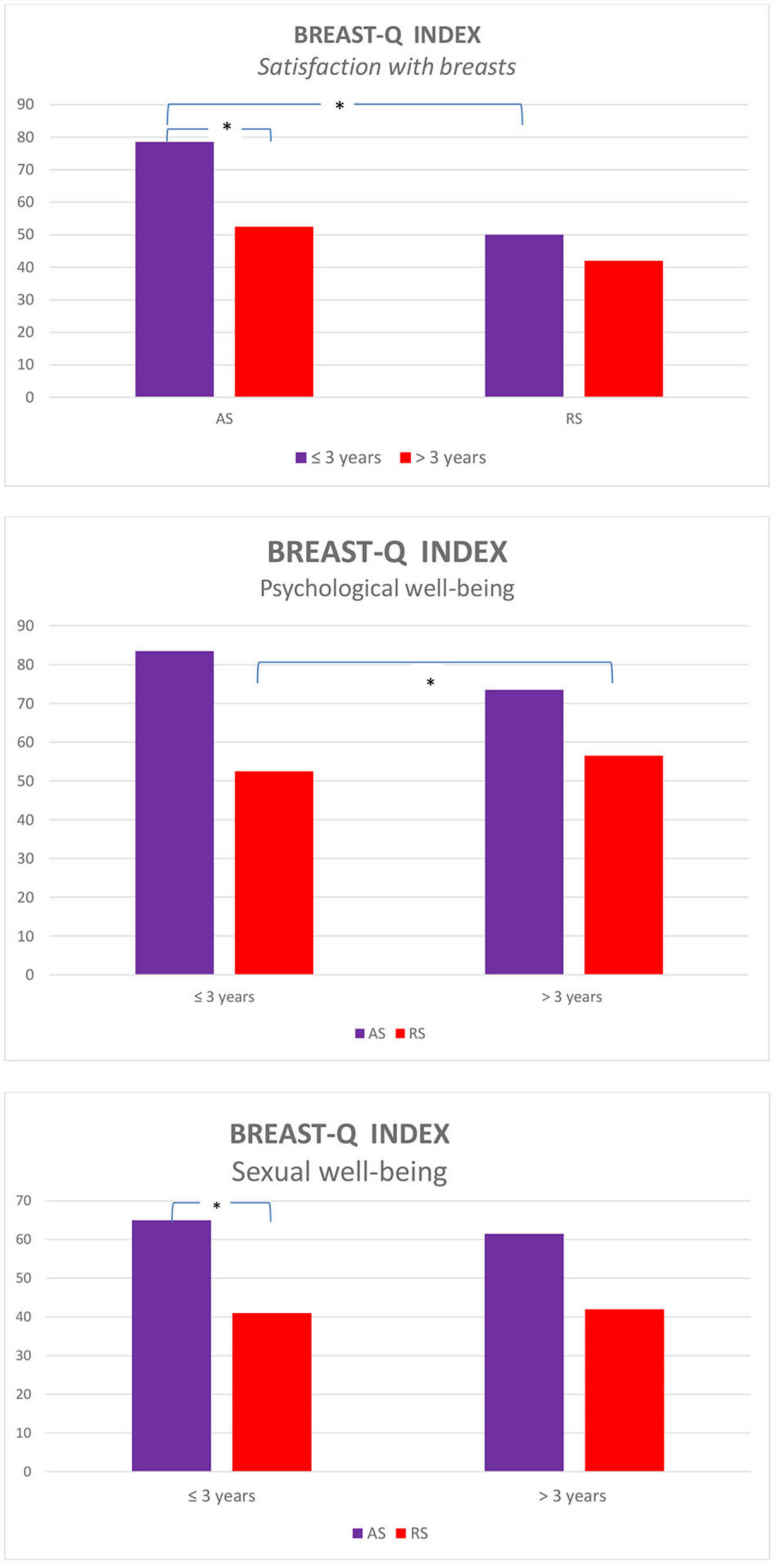
BREAST-Q indices for the time elapsed since the surgery between the augmentation surgery (AS) and reconstructive surgery (RS) groups.

Figure 2 presents the BREAST-Q indices of AS and RS group participants.

## Discussion and Conclusions

This study examined the psychological well-being of women who had undergone mammoplasty. Moreover, we paid attention to the post-operative effect of the surgical interventions over a period of time. We analyzed the differences in the reactions and adaptation to the post-treatment changes to identify patterns in emotions. We examined the variables of post-surgery satisfaction and well-being by analyzing the psychological outcomes of two types of mammoplasty: aesthetic and disease-related plastic surgery.

The findings of several studies have underscored the positive effects of AS on women. Recently, Randquist et al. (19) highlighted the positive post-operative BREAST-Q results for AS, suggesting that the satisfaction with breasts is sustained in 70% of respondents. Positive changes were also observed in the other BREAST-Q indices. However, our findings showed that mammoplasty for aesthetic purposes can partially affect the well-being of women and lead to negative feelings over a period of time. Women who underwent AS ≤3 and >3 years earlier showed no differences in emotional functioning. However, differences were observed in their levels of satisfaction with breasts; this finding could explain the recent reduction in augmentation surgeries as revealed through the aesthetic plastic surgery databank (1). We compared two groups of women who differed in terms of the time that had elapsed since their surgery. There was a significant difference in the scores on the satisfaction with breasts subscale among the AS group participants who had undergone breast surgery much earlier; additionally, they had developed negative feelings toward their augmented breasts. These unexpected findings that suggest a decline in satisfaction among participants who had undergone breast AS, emphasize the need for in-depth investigations into the role of emotional regulation in facilitating the decision to opt for aesthetic surgery. These efforts should not only be mindful of the lack of self-acceptance that results from the need to conform to social norms and cultural standards but also consider the effectiveness of AS without integrated assistance. In this study, psychological and sexual well-being scores did not change over time. These findings suggest that the efficacy of breast AS is sustained only during the early post-operative phases. Future research studies measuring more structured emotional dimensions could lend stronger support to our findings by distinguishing the primary psychological aspects impacted by aesthetic surgery.

We examined the impact of mammoplasty on the satisfaction levels and well-being of women who had undergone RS (after MRM). In line with our hypothesis, women who belonged to the RS group were less satisfied with their breasts than those who belonged to the AS group. However, this was true only for those who had undergone surgery ≤3 years earlier. This finding suggests that their level of satisfaction with their breasts gradually declined, and these differences are attenuated and rendered insignificant over time. This finding is not surprising because women opt for RS after receiving a cancer diagnosis and undergoing mastectomy. Therefore, their decision to alter the appearance of their breasts is not guided by aesthetic preferences or psychological needs. In contrast, women opt for RS to rectify the ramifications of therapeutic surgeries (e.g., removal of cancer tissue). Oncological treatment has complex effects on the emotional functioning of patients (20, 21). Consequently, psychological interventions have proved to be effective in improving their post-treatment emotional functioning (22).

In this study, we focused on the post-operative psychological and sexual well-being after undergoing breast surgery. The results revealed that the time elapsed since their surgery appeared to be a predictor of negative feelings toward the changes in their breasts. Their impact on women's body image and perceived sexuality persists over time. Thus, psychological fragility, resulting from RS (after MRM), may act as a barrier, hindering their attempts to return to normalcy. Our findings highlight the psychological needs of oncological patients dealing with breast reconstructive surgery; these needs are not limited to the oncological diagnosis and related clinical treatment (pharmacological and non-pharmacological) but extend to the impact of bodily changes following plastic surgery, which affects the self-awareness, self-confidence, and psychological well-being in general.

There are some limitations in the present study: (a) the lack of psychological assessments for both groups before the surgical interventions; (b) absence of test–retest anxiety evaluation in the pre- and post-surgery phase; and (c) the lack of personality and emotional evaluations in order to detect and quantify mood disorders, quality of life, and psychological well-being. Our study initiated the investigation on this topic, while the limitations mentioned were rectified in ongoing studies by introducing new protocols. Preliminary analyses of collected data aid in designing and improving diagnosis procedures as well as tailored psychological interventions to deal with the breast (augmentation/reconstruction) surgery.

In conclusion, our findings underscore the need to provide psychological support to those who undergo breast AS and RS. Moreover, the provision of psychological support will ensure that patients do not experience psychological fragility after they undergo breast AS, although this may not remedy negative body image or self-perception. Breast AS can enhance psychological and sexual well-being, but it does not facilitate emotional adaptation or enhance satisfaction. The post-operative emotional functioning (e.g., self-perception, body image) of women who undergo RS tends to be non-optimal. However, over time, they adapt to the physical changes in their breasts. Therefore, to speed up this adaptation process and enhance their mental well-being, personalized psychological interventions should be made available. Additionally, tailored psychological support programs that help patients accept the appearance of their breasts and promote body positivity should be implemented immediately after RS.

## Data Availability Statement

The raw data supporting the conclusions of this article will be made available by the authors, without undue reservation.

## Ethics Statement

The studies involving human participants were reviewed and approved by Ethical approval to conduct this study was granted by the Institutional Review Board of the University of L'Aquila, Italy (Prot. No. 36032/2017), and S. Salvatore Hospital of L'Aquila (Italy) (i.e., the hospital from which the participants were recruited). Informed consent was obtained from each participant, and the study adhered to guidelines outlined in the Declaration of Helsinki (15). The patients/participants provided their written informed consent to participate in this study.

## Author Contributions

DD: conceptualized the study. FG and FF: elaborated study design. JR and EP: conducted the study. AM: supervisioned the study analysis. All authors contributed equally to the paper and approved final form.

## Conflict of Interest

The authors declare that the research was conducted in the absence of any commercial or financial relationships that could be construed as a potential conflict of interest.
